# Analgesic Effects of Compression at Trigger Points Are Associated With Reduction of Frontal Polar Cortical Activity as Well as Functional Connectivity Between the Frontal Polar Area and Insula in Patients With Chronic Low Back Pain: A Randomized Trial

**DOI:** 10.3389/fnsys.2019.00068

**Published:** 2019-11-13

**Authors:** Kanae Kodama, Kouichi Takamoto, Hiroshi Nishimaru, Jumpei Matsumoto, Yusaku Takamura, Shigekazu Sakai, Taketoshi Ono, Hisao Nishijo

**Affiliations:** ^1^Department of System Emotional Science, Faculty of Medicine, University of Toyama, Toyama, Japan; ^2^Department of Sports and Health Sciences, Faculty of Human Sciences, University of East Asia, Shimonoseki, Japan

**Keywords:** chronic low back pain, myofascial trigger point, prefrontal cortex, hemodynamic activity, oscillation, functional connectivity

## Abstract

**Background:**

Compression of myofascial trigger points (MTrPs) in muscles is reported to reduce chronic musculoskeletal pain. Although the prefrontal cortex (PFC) is implicated in development of chronic pain, the mechanisms of how MTrP compression at low back regions affects PFC activity remain under debate. In this study, we investigated effects of MTrP compression on brain hemodynamics and EEG oscillation in subjects with chronic low back pain.

**Methods:**

The study was a prospective, randomized, parallel-group trial and an observer and subject-blinded clinical trial. Thirty-two subjects with chronic low back pain were divided into two groups: subjects with compression at MTrPs (*n* = 16) or those with non-MTrPs (*n* = 16). Compression at MTrP or non-MTrP for 30 s was applied five times, and hemodynamic activity (near-infrared spectroscopy; NIRS) and EEGs were simultaneously recorded during the experiment.

**Results:**

The results indicated that compression at MTrPs significantly (1) reduced subjective pain (*P* < 0.05) and increased the pressure pain threshold (*P* < 0.05), (2) decreased the NIRS hemodynamic activity in the frontal polar area (pPFC) (*P* < 0.05), and (3) increased the current source density (CSD) of EEG theta oscillation in the anterior part of the PFC (*P* < 0.05). CSD of EEG theta oscillation was negatively correlated with NIRS hemodynamic activity in the pPFC (*P* < 0.05). Furthermore, functional connectivity in theta bands between the medial pPFC and insula cortex was significantly decreased in the MTrP group (*P* < 0.05). The functional connectivity between those regions was positively correlated with subjective low back pain (*P* < 0.05).

**Discussion:**

The results suggest that MTrP compression at the lumbar muscle modulates pPFC activity and functional connectivity between the pPFC and insula, which may relieve chronic musculoskeletal pain.

**Trial registration:**

This trial was registered at University Hospital Medical Information Network Clinical Trials Registry (UMIN000033913) on 27 August 2018, at https://upload. umin.ac.jp/cgi-open-bin/ctr/ctr_view.cgi?recptno=R000038660.

## Introduction

Chronic musculoskeletal pain is a substantial health concern, which may lead to worsening of daily living activities over time (Nakamura et al., 2011; Sugai et al., 2017). Chronic musculoskeletal low back pain is the most frequently reported chronic musculoskeletal pain (Nakamura et al., 2011). The myofascial trigger point (MTrP) is a painful spot in a taut band of skeletal muscle fibers. When compressed, the MTrP can produce referred pain, and it has been reported to be a main cause of chronic musculoskeletal pain (Simons et al., 1999a, b). An epidemiological study reported that ratios of the presence of MTrPs in the lumbar muscles in patients with chronic low back pain were higher than in subjects without low back pain (Iglesias-González et al., 2013). Compression at MTrPs has been reported to be an effective massage technique for acute and chronic musculoskeletal pain (Hou et al., 2002; Hains et al., 2010; Cagnie et al., 2013; Takamoto et al., 2015; Morikawa et al., 2017). It is noted that prolonged nociceptive inputs from MTrPs induce plastic changes in the brain, resulting in development and maintenance of chronic musculoskeletal pain (Niddam, 2009; Hocking, 2013; Shah et al., 2015). These findings suggest that analgesic effects of MTrP compression may be mediated through its effects on the central nervous system.

Recent neuroimaging studies have reported that chronic musculoskeletal pain induces morphological and functional activity alteration in the brain regions involved in somatosensory, emotional, and cognitive processing of pain including the prefrontal cortex (PFC), insula, anterior cingulate cortex, and somatosensory cortex (Coppieters et al., 2016; Niddam et al., 2017, 2008). Chronic pain was also shown to induce alteration of functional connectivity between these brain areas (Coppieters et al., 2016; Niddam et al., 2017, 2008). Brain oscillation, measured using electroencephalograms (EEGs), has been implicated in pain information processing in these brain areas (Sarnthein et al., 2005; Stern et al., 2006; Ploner et al., 2017). Various therapies for chronic pain have been shown to affect pain-related brain activity. MTrP compression and thermotherapy in the neck in patients with chronic neck pain was shown to suppress neck pain, as well as hemodynamic activity in the frontal polar PFC (Yasui et al., 2010; Morikawa et al., 2017). Furthermore, other treatments, such as acupuncture, physical therapies, and pharmacological therapies, have been shown to induce structural and functional activity changes, especially in the PFC, and also induce changes in functional connectivity among the pain-related brain areas in patients with chronic musculoskeletal pain (Baliki et al., 2008; Napadow et al., 2012; Li et al., 2014; Kregel et al., 2017). However, the neural mechanisms underlying the analgesic effects of MTrP compression in chronic low back pain remain unknown.

In the present study, we hypothesized that compression at lumbar MTrPs may affect hemodynamic and oscillatory activity in the PFC, as well as functional connectivity between the PFC and other pain-related brain areas, in patients with chronic musculoskeletal low back pain. To analyze these parameters, we used simultaneous EEG and near infrared spectroscopy (NIRS) recording.

## Materials and Methods

### Experimental Design and Subjects

The study was a prospective, randomized, parallel-group trial and an observer- and subject-blinded clinical trial. This trial was performed from 27 August 2018 to 18 January 2019 in university of Toyama. The experiment was carried out in accordance with the guidelines set by the Helsinki Declaration and was approved by the ethics committee of the University of Toyama. Before commencement, the study was registered in UMIN Clinical Trials Registry as UMIN 000033913. We obtained written informed consent from the all subjects. The structure of the present study was shown in Supplementary Table S1 based on The Consolidated Standards of Reporting Trials (CONSORT) statement^1^.

**FIGURE 1:**
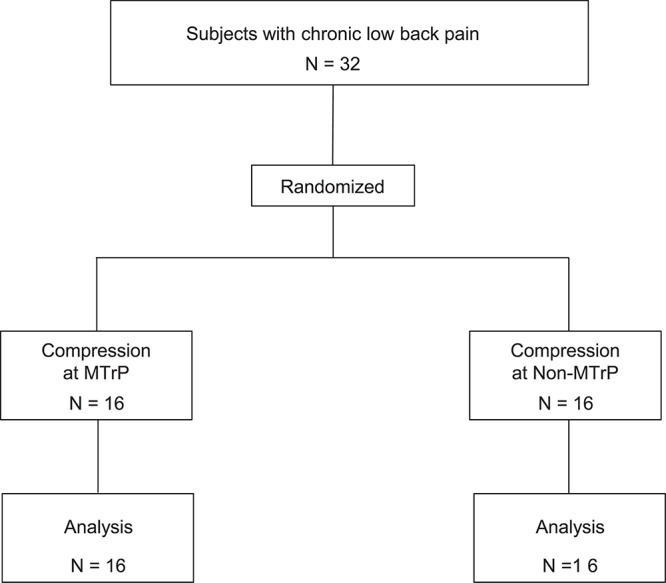
Participant flow diagram.

Thirty-two subjects with chronic low back pain (mean age 24.0 ± 0.9 years old; 14 males and 18 females) were enrolled in University of Toyama in the present study. The inclusion criteria for the participants were as follows: (1) persistent pain in the lumbar quadrate muscle for more than 3 months, (2) presence of active MTrP(s) in the lumbar quadrate muscle, (3) baseline lumbago scores over 40 mm in the visual analog scale (VAS), and (4) no neurological signs or peripheral nerve complaints. One therapist with over 6 years of clinical experience, who had the license for Japanese Judo therapy, identified active MTrPs according to the criteria for active MTrP diagnosis (Gerwin et al., 1997): (1) presence of hypersensitive spot(s) in taut band(s) in the muscle, and (2) reproduction of the same low back pain with the same referred pain as subjects usually felt when active MTrP(s) were stimulated by compression. The therapist detected the active MTrP in the lumbar quadrate muscle from all subjects (see below). The subjects were randomly assigned to two groups: the MTrP (compression at active MTrP) or non-MTrP (compression at non-MTrP) groups (Figure 1).

Active MTrPs in the lumbar quadrate muscle were identified according to the previous studies (Simons et al., 1999b; Joseph, 2009). First, the erector muscles of spine, iliac crest, and 12th rib were identified by palpation while the subjects lay on the bed in a prone position. Second, the lumbar quadrate muscle was identified by palpating the external edge of the erector muscles of spine, where the lumbar quadrate muscle is located. Finally, the therapist palpated the lumbar quadrate muscle in the area between the 12th rib and its iliac attachment to identify active MTrPs. The therapist usually identified active MTrPs in the lumbar quadrate muscle below the 12th rib in the present study.

### Sample Size

The sample size was computed on the basis of the previous clinical experiment that investigated effects of compression at active MTrPs and non-MTrPs on chronic musculoskeletal pain (Morikawa et al., 2017). In the previous study, the standard deviation of VAS score of pain at baseline was 9.8. The difference in VAS pain changes before and after treatment between the MTrP and non-MTrP groups was 14.2. Based on a significance level of 5% (two-tailed) and a statistical power of 95%, 14 subjects would be required in each group. With consideration of drop out and withdrawal rate, the adequate sample size was determined as 16 subjects in each group for the present study.

### Experimental Procedures

The subjects rated low back pain before experiment using the VAS score [from 0 (no pain) to 100 (maximum tolerable pain)] (primary outcome). Then, the subjects sat in a massage chair, were instructed to close their eyes, and the heads of the subjects were fitted with a combined NIRS/EEG head cap for simultaneous measurement of NIRS and EEG (Takeuchi et al., 2009) (secondary outcomes). The NIRS method allows hemodynamic measurements under a situation similar to clinical practice where subjects sit on a comfortable chair, and physicians can easily and exactly manipulate the low back muscle by hand. Then, 32 EEG electrodes and 61 NIRS probes were attached to the head cap. The pain pressure threshold (PPT) (secondary outcome) and maximal pressure pain threshold (MPT) in the stimulation site were measured using a digital algometer prior to the compression experiment (see the section “Muscle Compression (Intervention)” in detail).

The compression experiment was performed after a 5 min rest period while EEGs and NIRS were recorded as baseline activity. Then, active MTrP or non-MTrP compression was applied for 30 s, followed by a rest period of 60 s. This cycle was repeated five times (five cycles). The therapist kept predetermined constant pressure at an intensity midway between the PPT and MPT according to monitoring of the pressure intensity on the thumb. After the end of the experiment, the EEG and NIRS were further measured for 5 min without muscle compression. Then, PPT was similarly assessed three times, and the head cap was removed. Finally, comfort/discomfort score and pain intensity score during compression, as well as present low back pain after compression experiment, were assessed using the VAS. For the comfort/discomfort score, the participants were instructed to rate pleasantness of the compression after each cycle based on a scale of −10 to 10, where −10 and 10 represented the most unpleasant and pleasant experiences, respectively. For the pain intensity score, the participants were asked to rate the intensity of compression after each cycle based on a scale of 0 to 100 [from 0 (no pain) to 100 (maximum tolerable pain)].

### Muscle Compression (Intervention)

Before the experiment, the therapist identified each point as MTrP or non-MTrP in the lumbar quadrate muscle stimulation site. Non-MTrP was located at the point 3 cm away from the MTrP, where compression did not elicit local or referred pain, and no taut band was detected.

To determine compression intensity at each stimulation site, the therapist measured PPT and MPT by compression at the MTrP or non-MTrP using a digital pressure sensor (6 mm diameter) (PDA-2MPA: Tokyo Sokki Kenkyujo, Japan) that was attached on the therapist’s thumb and connected to a data collection hardware device (PCD-301B: Kyowa Dengyo, Japan). Pressure intensity was observed online via a monitor (DCS-100A: Kyowa Dengyo, Japan) that neither the therapist nor the subject could look at. An experimenter (therapist, Exp 1), who could not observe the monitor recording, determined the PPT and MTP according to the reports of the subject. Another experimenter (Exp 2) recorded compression intensity from the monitor. While Exp 1 gradually increased compression intensity at an MTrP or non-MTrP, the subjects were instructed to say “yes” when they initially felt pain [score 4 (pain threshold: PPT); score 0 (no pain)], and also when they felt severe pain [score 10 (severe pain)] (i.e., MTP). Exp 2 recorded compression intensity at PPT and MTP. The PPT and MTP were measured three times in each stimulation site, and midway, intensities between the mean PPT and mean MTP were determined for each subject.

### Randomization and Blinding

The subjects were randomly assigned to two groups (MTrP and non-MTrP). An independent operator, who was different from the therapist and experimenter, generated random allocation sequence (block randomization; block size, 2; allocation ratio, 1:1) using R software (ver. 3.43) before starting the trial. The operator sealed the allocation code in sequentially numbered opaque envelopes. The envelopes were opened in numerical order, and the subjects were automatically assigned to one of the two groups based on the allocation code. After the trial was completed, the operator confirmed that the subjects were exactly assigned according to the random allocation sequence.

The subjects were not informed whether MTrP or non-MTrP was compressed. An experimenter, who was unaware of the treatment groups, assessed VAS, PPT, MTP, comfort/discomfort score, and pain intensity score during compression. The experimenter was away from the experimental room while the therapist detected the stimulation sites (MTrPs and non-MTrPs).

### NIRS Recording

**FIGURE 2:**
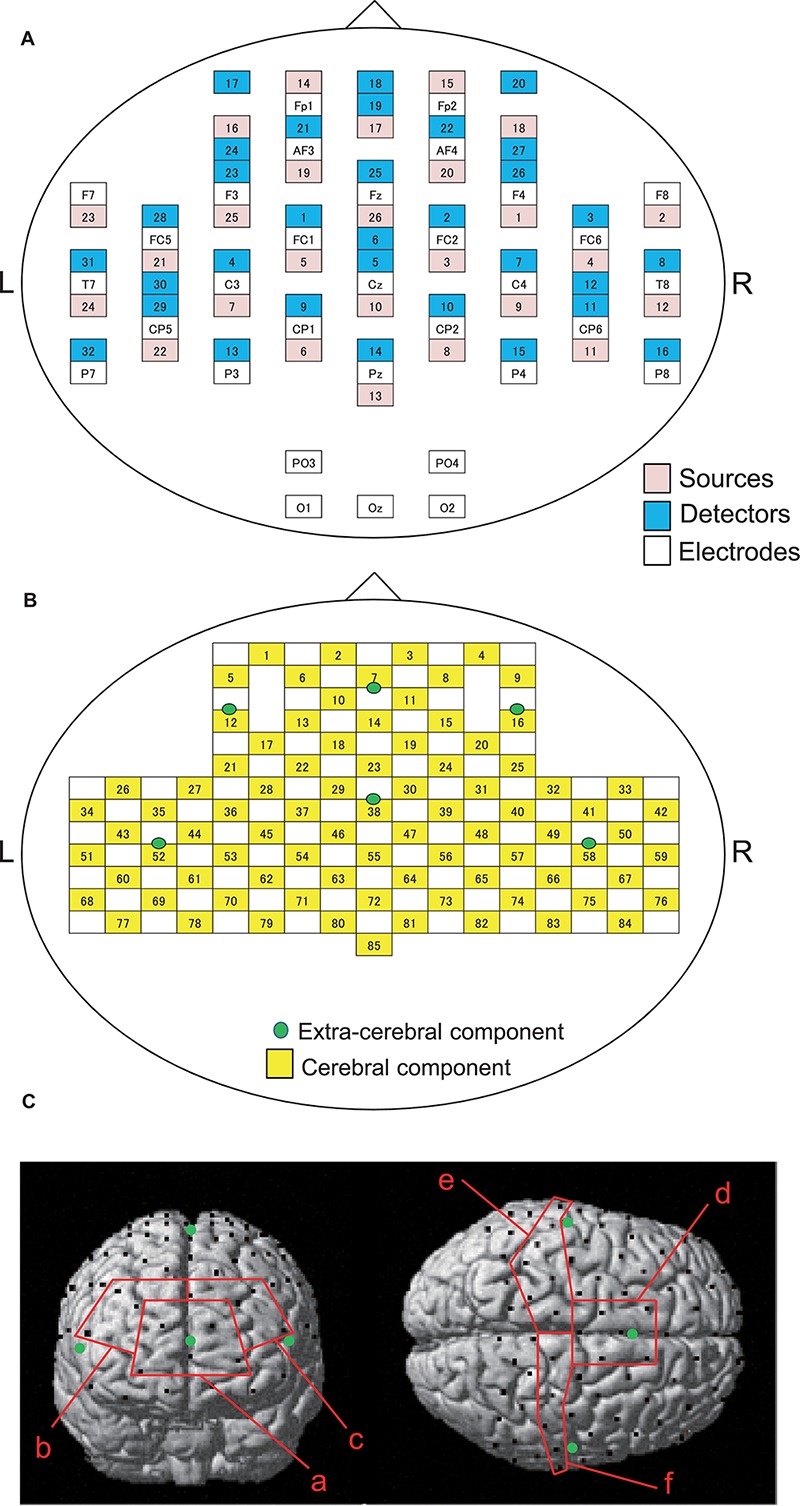
Arrangements of NIRS probes and EEG electrodes. **(A)** Arrangement of NIRS probes (source and detectors) and EEG electrodes. **(B)** Locations of NIRS channels. Green cycles indicate extra-cerebral component channels. **(C)** Spatial registration of NIRS channel locations. Black circles, NIRS channels; green circles, extra-cerebral component channels. (a) frontal polar area in the prefrontal cortex (pPFC); (b) right dorsolateral prefrontal cortex (R-dlPFC); (c) left dorsolateral prefrontal cortex (L-dlPFC); (d) supplementary motor area (SMA); (e) left primary sensorimotor cortex (L-SMC); (f) right primary sensorimotor cortex (R-SMC).

Cerebral hemodynamics were measured using two NIRS systems (OMM3000, Shimadzu Inc., Kyoto) in the present study. We used a head-cap for simultaneous recording of EEG and NIRS (FLASH-PLUS, Shimadzu Co. Ltd., Kyoto) (Takeuchi et al., 2009). The head cap was placed so that the bottom horizontal line of the NIRS optodes was positioned at the FP1-FP2 line of the 10–20 EEG system (Jurcak et al., 2007; Takeuchi et al., 2009). The head cap contained 26 source probes and 32 detector probes (Figure 2A). The hemodynamic response was detected by the near-infrared wave in the different three wavelengths (708, 805, and 830 nm) with 5 ms pulse. Hemoglobin concentration [oxy-Hb, deoxy-Hb, and total-Hb (Oxy-Hb + Deoxy-Hb)] was measured based on the modified Lambert-Beer law (Seiyama et al., 1988; Wray et al., 1988).

The information of the NIRS signals consists of both the cerebral and extra-cerebral hemodynamic components. The proportion of the two components depends on the distance between the source and detector probes (Fukui et al., 2003; Niederer et al., 2008). Hemodynamic signals from optodes with a distance over 3 cm reflect both cerebral and extra-cerebral (e.g., scalp, skull, etc.) components of the signals. When the distance of optodes is shorter than 1.5 cm, hemodynamic signals include mainly extra-cerebral components. Therefore, the optode arrangement with multi-distance was used to eliminate the interference from extra-cerebral hemodynamic components, including systemic changes of blood flow in the scalp layer and head motion artifacts, to the hemodynamic NIRS signals (Yamada et al., 2009, 2015; Saager et al., 2011; Ishikuro et al., 2014; Sato et al., 2016; Nakamichi et al., 2018).

In the present study, 26 detectors were placed 3 cm away from each source optode (Figure 2B). The midpoints between the source and detector optodes were defined as the “whole component channels,” and the signals from those optodes were defined as “whole signals.” This arrangement of the optodes resulted in 85 channels (and corresponding signals). In addition, another 6 detector optodes were placed 1.5 cm away from source optodes. The midpoints between the source and those detector probes were defined as “extra-cerebral component channels,” and the signals from those optodes were “extra-cerebral signals.” A total of six extra-cerebral signals were recorded from the corresponding extra-cerebral component channels (Figure 2B).

After the experiment in each subject, the three-dimensional coordinate positions of the optodes and channels was measured by a digitizer (Fastrak, Polhemus Inc., United States) with reference to the bilateral external auditory meatus and nasion.

### NIRS Data Analysis

The cerebral components of the NIRS signals (oxy-Hb, deoxy-Hb, total-Hb) were estimated using simple-subtraction methods (Schytz et al., 2009; Nakamichi et al., 2018). The cerebral components of the signals were estimated by subtraction of the extra-cerebral signals, located closest to corresponding whole signals, from the whole signals. Next, the resultant NIRS signals were band-pass filtered (0.01–0.1 Hz) to eliminate the baseline drift and physiological noises due to respiratory and cardiac activities (Tong et al., 2011; Yasumura et al., 2014).

Then, the NIRS signals during the five experimental cycles were summed and averaged to analyze the temporal changes of hemodynamics during the compression. The averaged hemodynamic responses were corrected for the mean baseline activity from −10 to 0 s before the compression. The change in Oxy-Hb concentration, which is the index most reflecting the neuronal activity (Hoshi et al., 2001), was converted to the effect size to compare the changes in hemodynamics between the two groups. The effect size of each channel in each cycle was calculated as [(averaged oxy-Hb concentration during compression for 30 s) - (averaged oxy-Hb concentration in the rest period from −10 to 0 s before the compression onset)]/the standard deviation of mean oxy-Hb concentration in the rest period. Then, the effect size from all five cycles was summed and averaged.

To identify the anatomical location of each channel, the three-dimensional coordinates of the channels in each subject were normalized to the Montreal Neurological Institute (MNI) coordinates by virtual registration (Tsuzuki et al., 2007). The anatomical locations [Brodmann’s areas (BA)] of the NIRS channels was estimated using the Talairach Daemon program^2^. We analyzed a total of six regions of interest (ROIs): frontal polar area in the PFC (pPFC; BA10), left and right dorsolateral prefrontal cortex (dlPFC; BA9,46), medial part of BA 6 that corresponds to the supplementary motor area (SMA), left sensorimotor cortex (L-SMC; BA1, 2, 3, and 4), and right sensorimotor cortex (R-SMC; BA1, 2, 3, and 4) (Figure 2C). Then, the effect sizes of the NIRS signals in the all channels in each ROI were summed and averaged.

### EEG Recording

EEG signals were recorded using a BioSemi ActiveTwo System (BioSemi CO. Ltd., Netherlands). After the head cap was placed on the head, 32 high-impedance electrodes, 2 reference electrodes, and grand were slotted into the holes in the head cap. Extra-electrodes for re-referencing were attached on the nose and both earlobes. To detect blinking and eye movements, electrooculograms (EOGs) were also recorded. It is noted that the EEG electrodes were placed at the midpoint between the NIRS source and detector optodes (Figure 2B), indicating that the locations of the EEG electrodes match those of the NIRS channels (Takeuchi et al., 2009). Electrode offset, which corresponds to input impedance, of all the electrodes were set to less than ±5 mV (Metting van Rijin et al., 1990). EEG signals were digitized using a 24-bit A/D converter at 2000 Hz sampling rate.

### EEG Data Analysis

The EEG data were preprocessed by using MATLAB (Mathworks Inc.) and the EEGLAB toolbox (Delorme and Makeig, 2004). The EEG data were down sampled from 2000 to 500 Hz and band-pass filtered in the range of 1–50 Hz. Then, EEG signals were removed automatically or by visual inspection if they were above ±50 μV or contained motion artifacts. Furthermore, EEG artifacts caused by eye movements and blinking were removed by using independent component analysis (ICA; Makeig et al., 1997, 1999; Jung et al., 2000; Delorme and Makeig, 2004). The artifact-free data of the 28 s period during each compression and 10 s period during rest after each compression were extracted from the EEG data.

Current source density (CSD) of neuronal oscillations was estimated from the EEG data using sLORETA (Pascual-Marqui, 2002). The solution space was confined to cortical gray matter, which consists of 6239.5 mm^3^ voxels. The artifact-free EEG data during compression (28 s for each cycle) and rest (10 s for each cycle) were segmented into 2 s epochs. A total of 70 and 25 of the 2 s epochs during compression and rest, respectively, in each subject were subjected to sLORETA analysis. Cross-spectra of the EEG epochs for each subject in each condition were computed using the sLORETA software in eight frequency bands: delta, 1.5–6.0 Hz; theta, 6.5–8.0 Hz; alpha1, 8.5–10.0 Hz; alpha2, 10.5–12.0 Hz; beta1, 12.5–18.0 Hz; beta2, 18.5–21.0 Hz; beta3, 21.5–30.0 Hz; and gamma, 31.0–50.0 Hz. The sLORETA computed the cortical distribution of CSD of neuronal oscillations in 6239 voxels from the averaged cross-spectra in each band for each subject in each condition. The CSD data were normalized with subject-wise normalization; CSD at each voxel was normalized with power density averaged across all frequencies and all 6239 voxels (Babiloni et al., 2007). Then, subtracted CSD (compression – rest) (defined as “CSD responses” during compression) were compared between the two groups by voxel-by-voxel in each band using the *t*-statistical non-parametric mapping (SnPM) implemented in sLORETA.

The significant voxels were counted in each brain region defined in sLORETA (see Table 3 in section “Results”). Since Brodmann area (BA) 10 includes a large cortical region, it was divided into two subregions in each side based on the map in sLORETA: mpPFC (a medial part of BA 10; i.e., BA 10 area in the medial frontal gyrus in sLORETA) and the remaining lateral part of BA 10 (lpPFC; i.e., BA10 area in the superior, middle and inferior frontal gyri, and subgyrus). BA 11 (OPFC in sLORETA) was also divided into two subregions: superior OPFC (an anterior part of BA 11; i.e., BA11 area in the superior frontal gyrus in sLORETA) and the remaining other region (rOPFC) in BA11 (BA 11 area in the inferior frontal; medial frontal, middle frontal, orbital, rectal, and subcallosal gyri in sLORETA). The dlPFC included BA 9 and 46 in sLORETA. The primary somatosensory cortex included BA 1, 2, and 3 in sLORETA. The insula cortex was divided into two regions: the insula in BA 13 (INS13) and the remaining other regions (oINS). The remaining other regions (oINS) included right BA22, right BA40, right BA41, left BA41, left BA45, and right BA47.

### Relationships Between EEGs and Hemodynamics

Relationships between CSD responses and effect sizes of hemodynamic responses (Oxy-Hb concentration) during compression were analyzed. First, brain regions that showed more than 30 voxels with significant differences in CSD responses between the two groups were identified in each frequency band in sLORETA (see above). In the present study, significant differences in CSD responses between the two groups were found only in theta bands (see section “Results”). Since spatial resolution of the NIRS is low, the right and left sides of each medial prefrontal region (mpPFC and lpPFC; superior OPFC and rOPFC) were each combined into one region. Then, mean CSD responses in theta bands in each brain region were computed by averaging CSD responses in the all voxels in each selected brain region. Then, mean theta CSD responses were subjected to natural logarithmic transformation to obtain a normal distribution. Finally, relationships between mean CSD responses and mean effect sizes of hemodynamic responses were analyzed in each selected brain region using a simple linear regression.

### Functional Connectivity

The 22 pain-related brain regions were selected as ROIs based on the map in sLORETA (Table 1). The ROIs included the right and left primary somatosensory cortex (S1) (BA 1, 2, and 3), right and left primary motor cortex (M1) (BA 4), right and left SMA, right and left dlPFC (BA 9, 46), right and left mpPFC, right and left lpPFC, right and left superior OPFC (BA11), right and left rOPFC (BA 11), right and left INS13 (BA 13 in the insula cortex), right and left v-ACC (BA 24), and right and left d-ACC (BA 32). According to a previous study (Cui et al., 2014), a single voxel in the center of each ROI was selected as a representative voxel in each ROI to compute functional connectivity since the spatial resolution of sLORETA was low.

**TABLE 1:** Cerebral regions of interest used for the lagged phase synchronization analysis.

**Anatomical regions**	**Brodmann area**	**ROI centroid MNI coordinates**					
		**Right**			**Left**		
		***X***	***Y***	***Z***	***X***	***Y***	***Z***
Primary somatosensory cortex (S1)	1,2,3	45	−30	50	−40	−30	50
Primary motor cortex (M1)	4	35	−25	50	−35	−20	50
Medial part of BA 6 (SMA)	6	30	−5	55	−30	−5	55
Dorsolateral prefrontal cortex (dlPFC)	9,46	30	35	30	−30	30	35
Medial part of frontal polar area (mpPFC)	10	10	55	5	−5	60	10
Lateral part of frontal polar area (lpPFC)	10	25	55	5	−25	55	5
Superior orbital prefrontal cortex (superior OPFC)	11	15	55	−20	−15	55	−20
Other part of orbital prefrontal cortex (oOPFC)	11	20	40	−20	−20	40	−20
BA 13 in the insula cortex (INS13)	13	40	−5	10	−40	−10	10
Ventral anterior cingulate cortex (v-ACC)	24	5	0	35	−5	0	35
Dorsal anterior cingulate cortex (d-ACC)	32	5	30	20	−5	30	20

To estimate functional connectivity between all possible pairs of ROIs based on sLORETA, lagged phase synchronization was used (Pascual-Marqui, 2007a, b; Canuet et al., 2011). This measure (lagged phase synchronization) evaluates similarity (nonlinear functional connectivity) between signals in the frequency domain according to normalized Fourier transforms and is corrected with the instantaneous zero-lag contribution. The instantaneous zero-lag contribution often includes non-physiological components such as volume conduction or physical artifact. This measure (lagged phase synchronization) is thought to contain only connectivity information without non-physiological components (Pascual-Marqui, 2007a, b; Canuet et al., 2011).

The independent sample *t* test and non-parametric randomizations (number of randomizations = 5000) were used to analyze significant difference in functional connectivity between all the possible pairs of ROIs in eight frequency bands between the MTrP and non-MTrP groups (Nichols and Holmes, 2002).

### Statistical Analysis

All data in the present study were shown as the average ± standard error. The Kolmogorov–Smirnov test was used to assess normality distribution for each continuous variable. Comparison of the baseline characteristics (age, VAS, PPT, and intensity of compression) and sensations evoked by compression (comfort/discomfort scores and pain intensity score during compression) between the two groups were performed by Student’s *t* test. The gender and stimulation site between the two groups were compared by Fisher’s exact test. Comparisons of changes in VAS and PPT before and after compression between the MTrP and non-MTrP groups were performed using Mann–Whitney *U* test. The effect sizes of hemodynamic responses during compression were compared by a repeated-measures two-way ANOVA (group × ROI). Relationships between effect sizes of hemodynamic responses (Oxy-Hb) and mean CSD in each band in each ROI were analyzed using the simple regression test. All the statistical analyses, except those implemented in sLORETA, were conducted using SPSS 19.0 (IBM Inc., New York, United States). Statistically significant levels were set to values of *P* < 0.05.

## Results

### Baseline Characteristics

Table 2 shows baseline clinical characteristics, stimulus condition, and sensations evoked by compression in each group. There were no significant differences between the two groups in age, VAS, PPT, intensity of stimulation, comfort/discomfort scores, or pain intensity scores during compression (Student’s *t* test, *P* > 0.05) and gender (Fisher’s exact test, *P* > 0.05). Furthermore, there was no significant difference in laterality of stimulation sites between the two groups (Fisher’s exact test, *P* > 0.05).

**TABLE 2:** Baseline characteristics and stimulus conditions in the two groups.

	**non-MTrP**	**MTrP**
	**(*n* = 16)**	**(*n* = 16)**
Age (years)	23.2 ± 0.6	24.6 ± 1.6
Stimulation side (R/L)	12/4	6/10
VAS (mm)	53.2 ± 2.6	55.4 ± 3.1
PPT (kPa)	128.9 ± 12.6	103.6 ± 6.6
Intensity of compression (kPa)	154.4 ± 14.2	131.6 ± 7.1
Pain intensity score during compression	56.5 ± 3.8	52.7 ± 3.9
Comfort/discomfort score during compression	51.1 ± 3.6	51.6 ± 3.4

*Data are presented as mean ± SE. R/L, right side/left side; VAS, visual analog scale; PPT, pressure pain threshold.*

### Changes in VAS Pain and PPT by Compression

**FIGURE 3:**
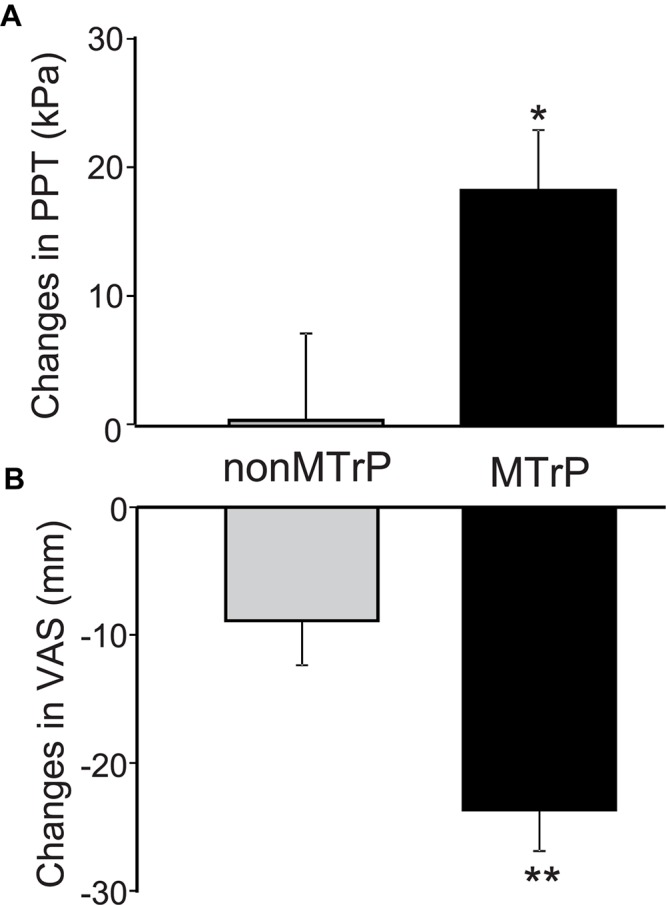
Changes of PPT **(A)** and VAS scores of low back pain **(B)** before and after compression in the MTrP and non-MTrP groups. Note that compression significantly increased PPT **(A)** and decreased VAS pain **(B)** in the MTrP group compared with the non-MTrP group (*P* < 0.05).

Compression significantly affected PTT and VAS pain. Figure 3A shows a PPT increment after compression in the two groups. Changes of PPT before and after compression was significantly increased after compression in the MTrP group [18.35 ± 4.69, mean ± SEM; 95% confidence interval (CI), 8.03 to 28.68] than the non-MTrP group (0.42 ± 6.78; CI, -14.50 to 15.34) [Mann–Whitney *U* test, *P* < 0.05; effect size (*r*) = 0.36]. Figure 3B shows VAS pain reduction after compression in the two groups. Compression significantly decreased changes of VAS pain before and after compression in the MTrP group (−23.69 ± 3.21; CI, −30.75 to −16.62) than the non-MTrP group (−8.88 ± 3.47; CI, −14.50 to 15.34) [Mann–Whitney *U* test, *P* < 0.05; effect size (*r*) = 0.55]. No adverse incidents were observed in the two groups.

### Hemodynamic Responses

**FIGURE 4:**
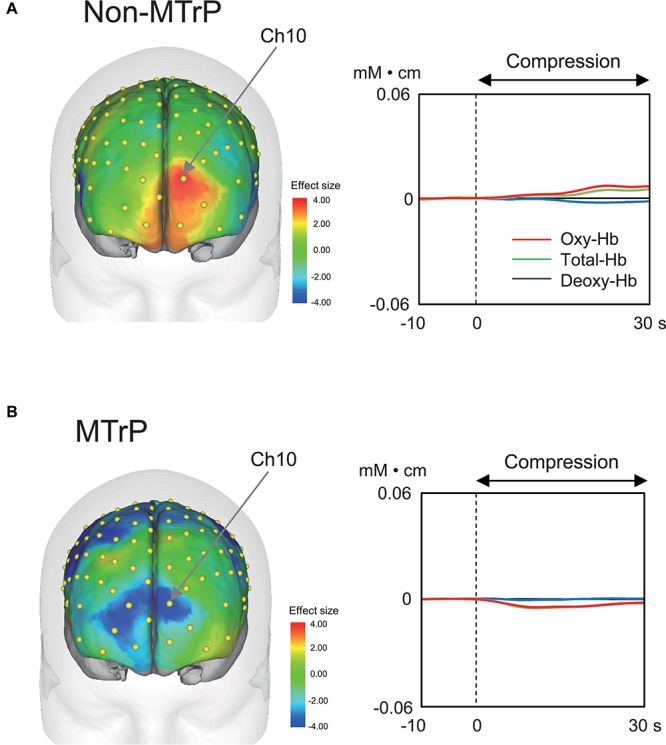
Examples of hemodynamic responses during compression in the non-MTrP **(A)** and MTrP **(B)** groups. Topographical maps in the left indicate effect sizes of hemodynamic responses (change in Oxy-Hb concentration) during compression. Yellow dots indicate NIRS channels. The right graphs indicate temporal changes in hemodynamic activity during compression.

Compression significantly affected cerebral hemodynamic activity. Figure 4 shows typical examples of hemodynamic responses during compression for 30 s at MTrPs and non-MTrPs, shown as effect sizes of Oxy-Hb concentration. The topographical maps of effect sizes indicated that hemodynamic activity increased in the pPFC during compression at a non-MTrP (left panel in A), while hemodynamic activity decreased in the pPFC during compression at an MTrP (left panel in B). Temporal patterns of Oxy-Hb signals showed similar changes; oxy-Hb signals gradually increased in the pPFC during compression at non-MTrPs (right panel in A), while Oxy-Hb concentration gradually decreased in the pPFC during MTrP compression (right panel in B).

**FIGURE 5:**
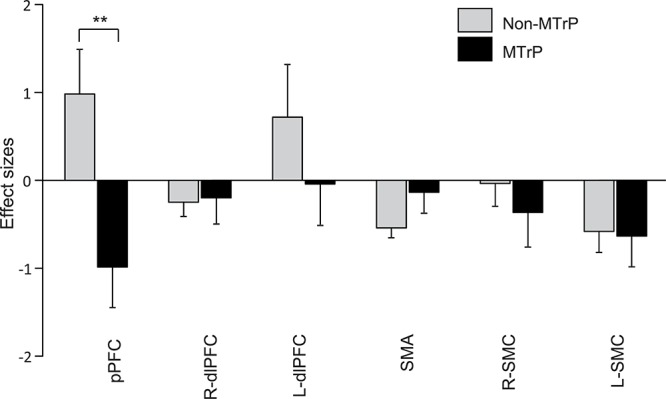
Comparison of hemodynamic responses in each ROI during compression between the MTrP and non-MTrP groups. Effect sizes of hemodynamic responses during compression was significantly decreased in the MTrP group compared with non-MTrP. pPFC, frontal polar area in the PFC; dlPFC, dorsolateral PFC; SMA, supplementary motor area; L-SMC, left primary sensorimotor cortex; R-SMC, right primary sensorimotor cortex.

Figure 5 shows the mean effect sizes of hemodynamic responses in the six ROIs in the MTrP and non-MTrP groups. The analysis by repeated-measures two-way ANOVA with “group” and “ROI” as factors showed no significant main effects of group (MTrP vs. non-MTrP) [*F*(1,30) = 1.61, *P* > 0.05] or ROI [*F*(2.96,88.77) = 2.02, *P* > 0.05], but showed a significant interaction between group and ROI [*F*(2.96,88.77) = 3.50, *P* < 0.05]. *Post hoc* comparisons revealed that Oxy-Hb concentration in the pPFC during compression was significantly decreased in the MTrP group compared to the non-MTrP group (Bonferroni test, *P* < 0.01).

### Brain CSD Activity

**FIGURE 6:**
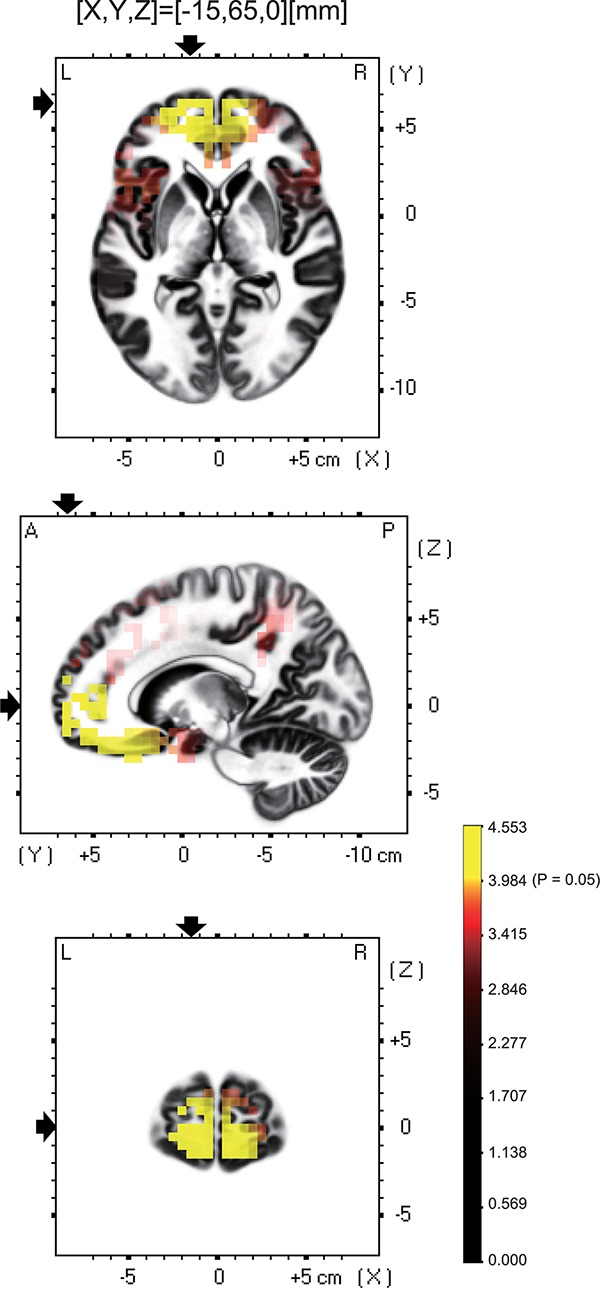
Brain maps showing significant changes in CSD of theta oscillations during compression between the MTrP and non-MTrP groups. Yellow areas show brain voxels with significantly higher theta CSD in the MTrP group compared with the non-MTrP group (*P* < 0.05). The black arrows indicate the 3D coordinates of the voxel with the highest CSD.

Among the eight frequency bands, we found significant differences only in CSD in theta bands between the MTrP and non-MTrP groups. Figure 6 shows significant differences in CSD between the MTrP and non-MTrP groups during compression. CSD in the theta bands increased mainly in the anterior part of the PFC during compression in the MTrP group than the non-MTrP group. Significant voxels were observed in the right primary somatosensory cortex (S1; BA 1–3), right primary motor cortex (M1;BA 4), bilateral supplementary motor cortex (SMA;BA 6), left frontal eye field (FEF;BA 8), bilateral dlPFC (BA 9, 46), bilateral mpPFC (BA 10), bilateral lpPFC (BA10), bilateral superior OPFC (BA 11), bilateral OPFC (BA 11), left middle temporal gyrus (MT; BA 21), bilateral subgenual cortex (BA 25), bilateral d-ACC (BA 32), left temporal pole (TP;BA 38), left opercular part of inferior frontal gyrus (OpIFG; BA 44), left triangular part of inferior frontal gyrus (TrIFG; BA 45), and left inferior frontal cortex (IFC) (BA 47) (Table 3).

**TABLE 3:** Number of voxels with significant differences in CSD of theta oscillations between the MTrP and non-MTrP groups in each brain area.

**Brain region**	**Brodmann**	**Number of**	
	**area**	**significant voxels**	
		
		**Right**	**Left**
Primary somatosensory cortex (S1)	1,2,3	2	0
Primary motor cortex (M1)	4	3	0
Medial part of BA 6 (SMA)	6	1	14
Frontal eye field (FEF)	8	0	1
Dorsolateral prefrontal cortex (dlPFC)	9,46	14	11
Medial part of frontal polar area (mpPFC)	10	18	31
Lateral part of frontal polar area (lpPFC)	10	9	39
Superior orbital prefrontal cortex (superior OPFC)	11	21	26
Other part of orbital prefrontal cortex (oOPFC)	11	37	88
BA 13 in insula cortex (INS13)	13	0	0
Other part of insula cortex (oINS)	22,40,41,45,47	0	0
Middle temporal gyrus (MT)	21	0	8
Ventral anterior cingulate cortex (v-ACC)	24	0	0
Subgenual cortex	25	1	4
Dorsal anterior cingulate cortex (d-ACC)	32	18	10
Temporal pole (TP)	38	0	49
Opercular part of inferior frontal gyrus (OpIFG)	44	0	10
Triangular part of inferior frontal gyrus (TrIFG)	45	0	4
Inferior frontal cortex (IFC)	47	0	38

*The number of voxels with significant power changes (*P* < 0.05) is listed for theta band.*

Among these brain regions, the bilateral mpPFC, bilateral lpPFC, bilateral superior OPFC, bilateral OPFC, left TP, and left IFC had more than 30 voxels with significant differences in CSD responses between the two groups.

### Relationship Between CSD and Hemodynamic Responses

**FIGURE 7:**
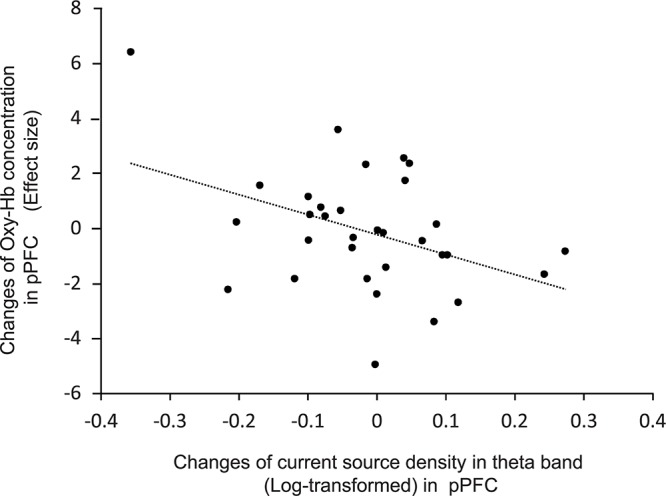
Relationships between changes in effect sizes of NIRS hemodynamic responses and changes in log-transformed CSD of EEG theta oscillations in the pPFC during compression.

Figure 7 shows the relationship between effects sizes of hemodynamic responses and logarithmic theta CSD responses during compression. The pPFC, OPFC, left TP, and IFC were selected as ROIs (see above section “Brain CSD Activity”). Statistical analyses by simple regression analysis revealed that the effect sizes of NIRS hemodynamic activity in the pPFC significantly and negatively correlated with the logarithmic theta CSD responses during compression in the pPFC [*r* = 0.41, *F*(1,30) = 5.88, *P* < 0.05]. In the other brain regions, there was no significant correlation between effect sizes of hemodynamic responses and logarithmic theta CSD responses (data not shown).

### Functional Connectivity During Compression

**FIGURE 8:**
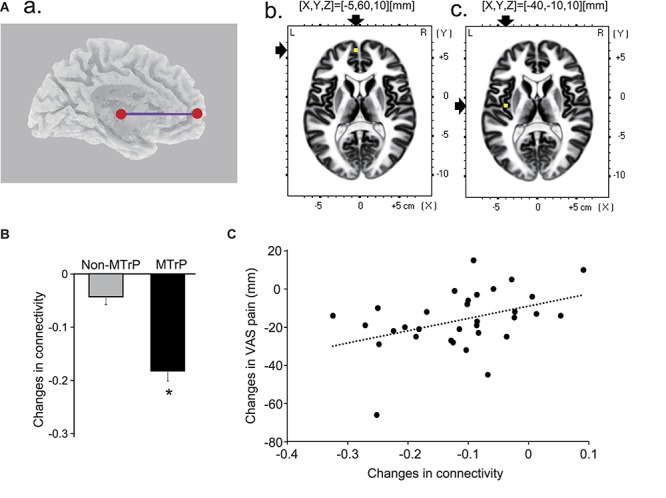
Changes in functional connectivity between the MTrP and non-MTrP groups during compression. **(A)** A purple line indicates a functional connectivity between the left mpPFC and left insula cortex (INS13) with significant reduction during compression in the MTrP group compared with the non-MTrP group. **(a)** Right two panels show the locations of the left mpPFC **(b)** and left insula cortex **(c)** in the MNI coordinates. **(B)** Comparison of changes in functional connectivity between the left mpPFC and insula cortex during compression between the non-MTrP and MTrP groups. ^∗^*P* < 0.05. **(C)** Relationships between functional connectivity changes shown in panel **B** and VAS pain changes during compression.

Although all possible pairs of ROIs were investigated in six frequency bands, there was only a significant difference in functional connectivity between the left mpPFC and left insula cortex in theta band between the MTrP and non-MTrP groups. Figure 8A shows functional connectivity in theta bands, which was significantly modulated by compression at the MTrP and non-MTrP. The statistical results in sLORETA indicated that only the functional connectivity between the left mpPFC and left INS13 was significantly different between the MTrP and non-MTrP groups (Figure 8A), and that this connectivity was significantly reduced during compression in the MTrP group than the non-MTrP group (Figure 8B). Furthermore, changes in functional connectivity in theta band between the left mpPFC and left INS13 by compression were significantly correlated with changes in VAS pain after compression [*r* = 0.41, *F*(1,30) = 5.97, *P* < 0.05] (Figure 8C).

## Discussion

### Effects of Compression on Cerebral Hemodynamics

In the present study, compression in the quadratus lumborum muscle improved low back pain and hyperalgesia and decreased hemodynamic activity in the pPFC in the MTrP group than the non-MTrP group. These results corroborate a previous NIRS study, in which compression at MTrPs in the trapezius muscle decreased hemodynamic activity in the pPFC in patients with chronic neck pain (Morikawa et al., 2017). Simultaneous NIRS and fMRI recording showed that the decrease in hemodynamic activity (Oxy-Hb concentration) was correlated with a decrease in blood oxygenation level-dependent (BOLD) signals in fMRI (Seiyama et al., 2004; Maggioni et al., 2015). It is reported that a decrease in BOLD signals reflects inhibition of neuronal activity (Huber et al., 2014; Sten et al., 2017). These findings suggest that compression at MTrPs inhibits neuronal activity in the pPFC regardless of locations of MTrPs involved in musculoskeletal pain.

The medial PFC including the pPFC (BA 10) is involved in sensory, emotional, and cognitive processing of pain, regulation of the autonomic nervous system, and pain chronification (McKlveen et al., 2015; Peng et al., 2018). Overactivation in the medial PFC was observed in patients with chronic low back pain (Baliki et al., 2006). PFC activity is correlated with cardiovascular sympathetic nervous activity during cognitive stress (Umeno et al., 2003) and muscle sympathetic nervous activity during acute tonic muscle pain (Kobuch et al., 2018). Taken together, the findings suggest that activity in the medial PFC, including the pPFC, increases in response to chronic pain, which in turn increases sympathetic nervous activity. We previously reported that MTrP compression in the neck decreased hemodynamic responses in the pPFC, the activity of which positively correlated with sympathetic nervous activity in patients with chronic neck pain (Morikawa et al., 2017). Furthermore, sympathetic nervous activity was positively correlated with subjective neck pain (Morikawa et al., 2017). It has been hypothesized that hyperactivity of sympathetic nervous activity induces the sustained muscle contraction and release of substances induced by muscle ischemia, which in turn develops MTrPs in the muscles (Gerwin, 2008). Thus, reduction of sympathetic nervous activity by MTrP compression through suppression of the medial PFC might induce a decrease in muscle tone and removal of algesic substances due to blood flow improvement, which, in turn, ameliorates chronic pain and hyperalgesia (Morikawa et al., 2017). The present results suggest that the same mechanisms are involved in reduction of chronic low back pain during compression at MTrPs in the quadratus lumborum muscle.

### Effects of Compression on EEG Oscillation

In this study, CSD of EEG theta oscillation increased more strongly in the anterior part of the brain including the pPFC in the MTrP group than the non-MTrP group. An EEG study reported that CSD of theta oscillations in the medial PFC was decreased during nociceptive stimulation and negatively correlated with pain intensity (Shao et al., 2012), whereas gamma oscillations in the PFC were positively correlated with subjective pain intensity in patients with chronic low back pain (May et al., 2019). Furthermore, some analgesic agents increased theta power (Malver et al., 2014). These results suggest that theta oscillations in the medial PFC are related to reduction in subjective pain.

In addition, CSD of theta oscillations in the medial PFC was negatively correlated with NIRS hemodynamic activity in the present study. Consistently, human non-invasive studies with simultaneous recordings of fMRI and EEG reported an inverse correlation between EEG theta oscillations and BOLD signals in the medial PFC (Mizuhara et al., 2004; Meltzer et al., 2007; Scheeringa et al., 2009; Michels et al., 2010; White et al., 2013). A theoretical study also suggests that neuronal activation increases BOLD signals and shifts the spectral profile of EEGs toward higher frequencies (Kilner et al., 2005). Taken together, the present EEG results also suggest that MTrP compression suppresses activity in the medial PFC including the pPFC, which may induce pain reduction.

Simultaneous EEG and ECG studies reported that an increase in theta oscillations in the PFC was associated with a reduction of sympathetic nervous activity or increment of parasympathetic nervous activity (Kubota et al., 2001; Takahashi et al., 2005; Tang et al., 2009). These results support the hypothesis that MTrP compression inhibits activity in the medial PFC, including the pPFC, which reduces sympathetic nervous activity. This reduction may in turn relieve chronic low back pain (see the above section “Effects of Compression on Cerebral Hemodynamics”).

### Effects of Compression on Brain Functional Connectivity

In the present study, functional connectivity based on theta coherence between the pPFC and insula cortex was significantly decreased in the MTrP group. Furthermore, functional connectivity between the pPFC and insula cortex positively correlated with subjective low back pain. It is suggested that the insula cortex plays an essential role in pain information processing including perception/sensation of pain, affective aspects of pain, maintenance, and progress of chronic pain, etc. (Benison et al., 2011; Cauda et al., 2011; Deen et al., 2011; Chang et al., 2013; Segerdahl et al., 2015), while the pPFC is also implicated in chronic pain (Apkarian et al., 2001; Baliki et al., 2006; Morikawa et al., 2017). Consistent with the current study, previous fMRI studies reported that functional connectivity between the insula cortex and default mode network (DMN) including the medial PFC increased in patients with chronic pain including chronic low back pain and fibromyalgia and correlated with subjective pain intensity (Cauda et al., 2009; Napadow et al., 2010; Loggia et al., 2013). Furthermore, pharmacological and physical interventions to reduce pain decreased such connectivity, while maneuvers to increase pain increased the functional connectivity (Napadow et al., 2012; Harris et al., 2013; Loggia et al., 2013). The current results, along with the previous fMRI studies, suggest that functional connectivity between the insula and other brain regions including the pPFC is one of the important determinants for development of chronic pain.

The pathway from the insula to the medial PFC is involved in information processing of uncontrollable pain (Bräscher et al., 2016), and the medial PFC may be involved in facilitation of pain sensation (Pastoriza et al., 1996; Lin et al., 2014). The present results indicated that coherence in the theta bands was decreased by manual compression. Synchronized activity between distant brain regions indicated by EEG coherence, especially in theta bands, reflects information transfer or memory retrieval between such distant brain regions (Jacobs et al., 2006; Saalmann, 2014). Furthermore, chronic pain is hypothesized to be associated with formation and activation of nociceptive memory (Yi and Zhang, 2011). Taken together, these findings suggest that manual compression at MTrPs may interfere with information flow of nociceptive memory from the insula to the pPFC by decreasing theta coherence. Consistent with this idea, functional connectivity between the medial PFC and insula cortex was involved in persistence of chronic low back pain (Hashmi et al., 2012).

The mechanisms of reduction of theta coherence are uncertain in the present study. However, changes in theta coherence could be attributed to changes in oscillations in the thalamus due to manual compression at MTrPs. Acupuncture stimulation of acupoints, 71% of which match trigger points anatomically (Melzack et al., 1977), activates Aβ-, Aδ-, and C-fibers (Chen et al., 2014). Most thalamic nuclei respond to stimuli activating these fibers (Ren et al., 2009; Cardoso-Cruz et al., 2013; Whitt et al., 2013; Chen et al., 2014; Bastuji et al., 2016). These findings suggest that compression at MTrPs modulates thalamic activity. On the other hand, the thalamus is proposed to coordinate cortical coherence (Saalmann, 2014), and the thalamic nuclei modulate cortical theta oscillations differently depending on the subnuclei of the thalamus (Sarnthein and Jeanmonod, 2008; LeBlanc et al., 2014, 2017). These findings further suggest that compression at MTrPs may reduce theta coherence between the pPFC and insula through its effects on the thalamus.

### Limitation

In the present study, we investigated effects of compression at active MTrPs on chronic low back pain. However, effects of compression at latent MTrPs are unknown in the present study. MTrPs are classified into active and latent MTrPs. Latent MTrPs are defined as follows: (1) presence of a hypersensitive spot in a taut band in a given muscle and (2) production of pain or referred pain that is not related to a given patient’s clinical pain complaint when latent MTrP(s) are stimulated by compression (Simons et al., 1999a, b). Levels of algesic substances and muscle stiffness were higher in active MTrPs than latent MTrPs (Shah and Gilliams, 2008; Shah et al., 2008; Calvo-Lobo et al., 2017). The number of active and latent MTrPs was greater in patients with chronic low back pain compared with healthy subjects (Iglesias-González et al., 2013). Furthermore, a previous study using patients with low back pain reported that muscle stiffness was higher while PPT was lower in lumbar areas with active and latent MTrPs compared with those with non-MTrP (Calvo-Lobo et al., 2017). These findings suggest that latent MTrPs could be also involved in low back pain. Further studies are required to investigate effects of compression at latent MTrPs in patients with chronic low back pain.

Second, palpation of the lumbar quadrate muscle is relatively difficult, compared with other muscles that are located superficially, since it could be palpated directly from the skin or indirectly below other muscles depending on its position (Simons et al., 1999b). Future studies such as those combined with elastography could more precisely diagnose active MTrPs in the lumbar quadrate muscle.

## Conclusion

The present study investigated the neural mechanisms of analgesic effects of MTrP compression in chronic low back pain using NIRS and EEG measurement. Both NIRS and EEG results indicated that MTrP compression significantly decreased activity in the pPFC, as well as decreased functional connectivity between the pPFC and insula cortex, the strength of which between those regions was negatively correlated with subjective low back pain. The results, along with the previous studies, suggest that MTrP compression at the lumbar muscle reduces coherent activity in the pPFC and insula cortex, which may decrease sympathetic activity involved in muscle pain.

MTrP compression did not affect gamma oscillations in the present study. However, previous human studies reported that an increase in gamma oscillations, especially in higher-frequency bands (50–120 Hz), was associated with pain perception in the PFC and somatosensory cortex (Zhang et al., 2012; Schulz et al., 2015; Li et al., 2016; Zhou et al., 2018; Hu and Iannetti, 2019; May et al., 2019). Thus, the absence of gamma oscillation changes could be ascribed to the low-frequency band (31–50 Hz) of gamma oscillations analyzed in this study. Furthermore, cross-frequency coupling between theta and gamma oscillations was increased during painful sensory stimulation or in a state with chronic inflammatory pain (Liu et al., 2015; Wang et al., 2016). The current results, along with these previous studies, suggest that high-frequency gamma oscillations coupled with theta oscillations is transferred from the insula cortex to the PFC, which may be involved in the perception of chronic pain. Reduction of functional connectivity in theta bands by MTrP compression may decrease this transfer of high-frequency gamma oscillation. Further studies to investigate higher-frequency gamma oscillations are required to address the role of functional connectivity between the pPFC and insula in chronic pain perception.

## Data Availability Statement

The data that support the findings in this study are available from the corresponding author HisN, upon reasonable request.

## Ethics Statement

The studies involving human participants were reviewed and approved by the ethics committee of the University of Toyama. The patients/participants provided their written informed consent to participate in this study.

## Author Contributions

HisN and KT designed the experiment. KK performed the experiment. KK, KT, and HisN analyzed the data and wrote the manuscript. HisN, HirN, JM, YT, SS, and TO revised the manuscript. All authors discussed the results, and approved the final manuscript.

## Conflict of Interest

The authors declare that the research was conducted in the absence of any commercial or financial relationships that could be construed as a potential conflict of interest.

## Footnotes

1 http://www.consort-statement.org/home/

2 www.talairach.org
