# Corrigendum: Analgesic Effects of Compression at Trigger Points Are Associated With Reduction of Frontal Polar Cortical Activity as Well as Functional Connectivity Between the Frontal Polar Area and Insula in Patients With Chronic Low Back Pain: A Randomized Trial

**DOI:** 10.3389/fnsys.2019.00081

**Published:** 2020-01-23

**Authors:** Kanae Kodama, Kouichi Takamoto, Hiroshi Nishimaru, Jumpei Matsumoto, Yusaku Takamura, Shigekazu Sakai, Taketoshi Ono, Hisao Nishijo

**Affiliations:** ^1^Department of System Emotional Science, Faculty of Medicine, University of Toyama, Toyama, Japan; ^2^Department of Sports and Health Sciences, Faculty of Human Sciences, University of East Asia, Shimonoseki, Japan

**Keywords:** chronic low back pain, myofascial trigger point, prefrontal cortex, hemodynamic activity, oscillation, functional connectivity

In the original article, there was an error. It was erroneously stated that the hemodynamic activity during compression at a non-MTrP decreased instead of increased and increased instead of decreased during compression at an MTrP.

A correction has been made to **Results, Hemodynamic Responses, paragraph one**:

“Compression significantly affected cerebral hemodynamic activity. Figure 4 shows typical examples of hemodynamic responses during compression for 30 s at MTrPs and non-MTrPs, shown as effect sizes of Oxy-Hb concentration. The topographical maps of effect sizes indicated that hemodynamic activity increased in the pPFC during compression at a non-MTrP (left panel in A), while hemodynamic activity decreased in the pPFC during compression at an MTrP (left panel in B). Temporal patterns of Oxy-Hb signals showed similar changes; oxy-Hb signals gradually increased in the pPFC during compression at non-MTrPs (right panel in A), while Oxy-Hb concentration gradually decreased in the pPFC during MTrP compression (right panel in B).”

The authors apologize for this error and state that this does not change the scientific conclusions of the article in any way. The original article has been updated.

